# Editorial: Open science in Africa

**DOI:** 10.3389/frma.2023.1233867

**Published:** 2023-06-14

**Authors:** Heila Pienaar

**Affiliations:** Department of Information Science, University of Pretoria, Pretoria, South Africa

**Keywords:** editorial, open science, Africa, open data, open access, policy, barriers and facilitative factors

The ongoing transition toward Open Science (OS) is increasing transparency and collaboration in the research enterprise. This Research Topic aims to investigate the transition to OS in Africa, including the concerns and advantages of OS for researchers and stakeholders. It also explores the role of new technologies and infrastructure in implementing OA and bridging the knowledge divide between countries. In this editorial, we provide an overview of eight articles that shed light on various aspects of open science, data sharing, and the challenges and opportunities they present in the African context. These articles highlight the importance of policymakers, institutions, and researchers working together to foster a culture of open science and to address the existing barriers to data accessibility on the African continent.

The article by Okafor et al. focuses on the adoption of open science (OS) practices in Africa, considering the limitations and prospects for its institutionalization. The authors emphasize the significance of science access for the advancement of scientific research and the development of the next generation of scientists in Africa. They highlight the global resurgence of discussions around open science due to the COVID-19 pandemic, particularly in resource-poor settings like Africa where OS practices are currently limited. Overall, the review article serves as an advocacy strategy and informative guide for policymakers and stakeholders involved in promoting and integrating open science practices in Africa. It highlights the importance of overcoming barriers and fostering a supportive environment for open science to thrive on the continent (Okafor et al.).

The next article, “*Rethinking the a in FAIR data: issues of data access and accessibility in research*” by Shanahan and Bezuidenhout, raises concerns about the assumptions of accessibility in FAIR (Findable, Accessible, Interoperable, and Reusable) data principles. The authors emphasize that access to FAIR data resources can be influenced by geopolitical factors, exacerbating existing access inequities. They stress the need for increased awareness and consideration of these issues in FAIR implementation (Shanahan and Bezuidenhout).

The article, “*Open science in Africa: what policymakers should consider*” by Chiware and Skelly, underlines the importance of African governments and institutions embracing open science principles and building research infrastructures that align with the global open science movement. The authors highlight the significance of OS policy frameworks and provide insights for policymakers, aiming to guide similar initiatives in Africa (Skelly and Chiware).

“*African researchers do not think differently about open data*” by Skelly and Chiware, explores African researchers' attitudes toward open data and demonstrates that their perspectives are not significantly different from their international counterparts. This finding emphasizes the need for policymakers and institutions to understand and address researchers' concerns and expectations regarding data sharing and the open data ecosystem (Skelly and Chiware).

In “*Open access and its potential impact on public health—a South African perspective*”, Strydom et al. examine the impact of open access on public health in South Africa. They highlight the benefits of open science and discuss financial implications and potential solutions for reducing publication costs for researchers and institutions. The authors also address privacy concerns and the role of data protection legislation in medical research and data reuse (Strydom et al.).

Hey's article, “*Open science and big data in South Africa*”, focuses on the challenges and opportunities presented by “Big Scientific Data” in South Africa, particularly in the context of the Square Kilometer Array project and the Multi-Purpose Reactor. The author highlights the importance of open science policies and the FAIR principles in managing and making such data accessible, proposing the use of semantic markup and emphasizing the role of interdisciplinary teams in research data management (Hey).

Chigwada's “*Feasibility of a national open data policy in Zimbabwe*” explores the potential for implementing a national open data policy in Zimbabwe. The study assesses the readiness of the country in terms of open data activities, highlighting the need for advocacy, awareness creation, and collaboration among stakeholders to craft and enact a national open data policy. The author emphasizes the value of government and research data for driving research and innovation (Chigwada).

“*Building awareness and capacity of bioinformatics and open science skills in Kenya: a sensitize, train, hack, and collaborate model*” by Karega et al., presents a framework for promoting bioinformatics and open science skills in Kenya. The authors showcase the Sensitize-Train-Hack-Collaborate/Community model, which combines awareness-building, training, collaborative projects, and community engagement to empower researchers with the necessary skills and tools in open science and bioinformatics (Karega et al.).

These articles collectively underscore the importance of open science, data accessibility, and policy development in Africa. They highlight the need for increased awareness, capacity building, and interdisciplinary collaborations to overcome challenges and leverage the potential of open science.

## Author contributions

The author confirms being the sole contributor of this work and has approved it for publication.

